# Overall survival with crizotinib and next-generation ALK inhibitors in *ALK*-positive non-small-cell lung cancer (IFCT-1302 CLINALK): a French nationwide cohort retrospective study

**DOI:** 10.18632/oncotarget.15746

**Published:** 2017-02-26

**Authors:** Michaël Duruisseaux, Benjamin Besse, Jacques Cadranel, Maurice Pérol, Bertrand Mennecier, Laurence Bigay-Game, Renaud Descourt, Eric Dansin, Clarisse Audigier-Valette, Lionel Moreau, José Hureaux, Remi Veillon, Josiane Otto, Anne Madroszyk-Flandin, Alexis Cortot, François Guichard, Pascaline Boudou-Rouquette, Alexandra Langlais, Pascale Missy, Franck Morin, Denis Moro-Sibilot

**Affiliations:** ^1^ Centre Hospitalier Universitaire Grenoble Alpes, Thoracic Oncology Unit, Chest Department, Grenoble, France; ^2^ Medical Oncology Department, Gustave Roussy, Villejuif, France; ^3^ Assistance Publique Hôpitaux de Paris, Tenon Hospital, Chest Department, Paris, France; ^4^ Léon-Bérard Cancer Center, Lyon, France; ^5^ Centre Hospitalier Universitaire de Strasbourg, Chest Department, Strasbourg, France; ^6^ Larrey Hospital, Chest Department, Toulouse, France; ^7^ Centre Hospitalier Universitaire de Brest, Brest, France; ^8^ Oscar Lambret Cancer Center, Medical Oncology Department, Lille, France; ^9^ Centre Hospitalier Sainte Musse, Chest Department, Toulon, France; ^10^ Centre Hospitalier Général de Colmar, Louis Pasteur Hospital, Chest Department, Colmar, France; ^11^ Centre Hospitalier Universitaire d’Angers, Chest Department, Angers, France; ^12^ Centre Hospitalier Universitaire de Bordeaux, Respiratory Disease Department, Pessac, France; ^13^ Antoine Lacassagne Cancer Center, Nice, France; ^14^ Paoli Calmettes Institute, Marseille, France; ^15^ Centre Hospitalier Universitaire de Lille, Thoracic Oncology Unit, Lille, France; ^16^ Medical Oncology Department, Polyclinique Bordeaux Nord Aquitaine, Bordeaux, France; ^17^ Assistance Publique Hôpitaux de Paris, Cochin-Port Royal Hospital, Medical Oncology Department, Paris, France; ^18^ French Cooperative Thoracic Intergroup, Department of Biostatistics, Paris, France; ^19^ French Cooperative Thoracic Intergroup, Clinical Research Unit, Paris, France

**Keywords:** lung cancer, ALK, crizotinib, ceritinib, alectinib

## Abstract

Overall survival (OS) with the anaplastic lymphoma kinase (ALK) inhibitor (ALKi) crizotinib in a large population of unselected patients with *ALK*-positive non-small-cell lung cancer (NSCLC) is not documented. We sought to assess OS with crizotinib in unselected *ALK*-positive NSCLC patients and whether post-progression systemic treatments affect survival outcomes.

**ALK:**

-positive NSCLC patients receiving crizotinib in French expanded access programs or as approved drug were enrolled. We collected clinical and survival data, RECIST-defined progressive disease (PD) and post-PD systemic treatment efficacy. We performed multivariable analysis of OS from crizotinib initiation and PD under crizotinib.

At time of analysis, 209 (65.7%) of the 318 included patients had died. Median OS with crizotinib was 16.6 months. The line of crizotinib therapy did not impact survival outcomes. Of the 263 patients with PD, 105 received best supportive care, 74 subsequent drugs other than next-generation ALKi and 84 next-generation ALKi. Next-generation ALKi treatment correlated with better survival outcomes in multivariate analysis. These patients had a median post-PD survival of 25.0 months and median OS from metastatic disease diagnosis of 89.6 months.

Unselected *ALK*-positive NSCLC patients achieve good survival outcomes with crizotinib therapy. Next-generation ALKi may provide survival improvement after PD under crizotinib.

## INTRODUCTION

The *anaplastic lymphoma kinase (ALK)* gene is rearranged in approximately 5% of non-small-cell lung cancer (NSCLC) cases, leading to constitutive activation of the ALK tyrosine kinase domain and tumorigenesis [1, 2]. Crizotinib is an inhibitor of ALK kinase activity that has demonstrated its superiority over conventional chemotherapy in advanced *ALK*-positive NSCLC [3, 4]. Crizotinib was compared to standard first-line and second-line chemotherapy in two randomized Phase III trials (PROFILE 1014 and PROFILE 1007), achieving higher response rates and a significantly longer median progression-free survival (PFS) [3, 4]. It is now approved worldwide for treating advanced *ALK*-positive NSCLC.

The estimation of overall survival (OS) with crizotinib has not yet been fully documented. A retrospective analysis comparing 30 crizotinib-treated *ALK*-positive NSCLC patients to 23 crizotinib-naïve ones reported longer OS in the former (1-year OS: 70% *vs*. 44%; 2-year OS: 55% *vs*. 12%, respectively) [5]. In the PROFILE 1007 trial comparing crizotinib to pemetrexed or docetaxel as second-line following platinum-based regimen failure, an updated survival analysis showed a median OS with crizotinib at 21.7 months but identified no difference in OS between the crizotinib arm and chemotherapy arm, probably due to a cross-over in the chemotherapy arm [6]. Another limitation comes from the restrictive inclusion criteria implemented in clinical trials, meaning any benefit observed in a selected population might not reflect that in daily practice.

Most patients experience progressive disease (PD) within one year of crizotinib initiation [3, 4]. Locally-ablative treatment could extend disease control with crizotinib in oligoprogressive disease [7, 8], and continuing crizotinib beyond PD (CBPD) might favourably impact survival outcomes [9]. Anecdotal clinical response to crizotinib rechallenge has been reported [10, 11]. The efficacy of conventional chemotherapy is debatable as very little clinical data is available after crizotinib failure [12].

Next-generation ALK inhibitors (ALKis) can overcome resistance to crizotinib. Ceritinib has proven efficacious in crizotinib-pretreated patients in a dose escalation Phase I trial (response rate: 56%; median PFS: 6.9 months), and has been approved by both FDA and EMA following crizotinib failure [13, 14]. Alectinib is also effective in crizotinib-pretreated patients (response rate: 48-55%; PFS: 8.1-8.9 months) and FDA approved [15–17]. Other promising next-generation ALKis are under clinical investigation [18, 19]. Given their efficacy, a potential survival benefit associated with using next-generation ALKi following crizotinib failure has been suspected, though not yet proven, in comparison with other systemic treatment options [20–22].

The IFCT-1302 CLINALK study sought to evaluate OS under crizotinib in a large unselected population of *ALK*-positive NSCLC patients. We also aimed to examine the different systemic treatments’ effect on survival following crizotinib and whether next-generation ALKis improve survival outcomes in this setting.

## RESULTS

### Patient characteristics

**Figure 1:**
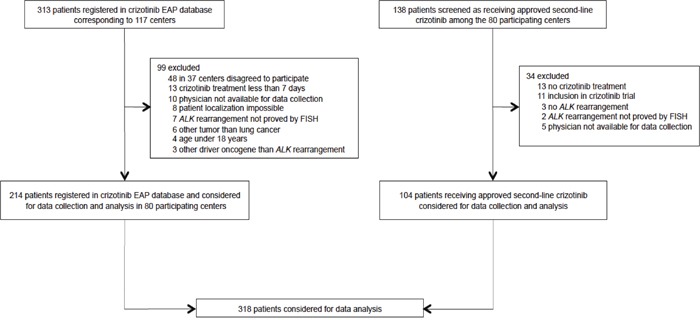
Study flow chart

In total, 318 patients were selected for data collection and analysis (Figure 1). Of them, 214 met the inclusion criteria in the French crizotinib expanded access program (EAP) database. Following EAP discontinuation, 104 additional *ALK*-positive patients treated with crizotinib as second-line approved drug were enrolled.

Table 1 presents the patients’ characteristics on initiating crizotinib. Half were male, the median age was under 60 years old, and the majority had never smoked and presented with adenocarcinomas. One third exhibited brain metastasis, and 77.3% PS 0 or 1.

**Table 1:** Baseline characteristics for the population at the time of crizotinib initiation

Characteristics, n (%)	N=318
Age, years	
Median (range)	58.3 (19.2–88.4)
<65	221 (69.5%)
≥65	97 (30.5%)
Gender	
Male	161 (50.6%)
Female	157 (49.4)
Ethnicity	
Non-Asian	294 (98.3%)
Asian	5 (1.7%)
MD	19
Smoking status	
Current-smoker	29 (9.4%)
Former-smoker	108 (34.8%)
Never-smoker	173 (55.8%)
MD	8
Histology	
Adenocarcinoma	292 (91.8%)
Large cell	19 (6.0%)
Other	7 (2.2%)
ECOG PS	
0	92 (31.6%)
1	133 (45.7%)
2	43 (14.8%)
3	21 (7.2%)
4	2 (0.7%)
MD	27
Stage at diagnosis	
Localized	5 (1.6%)
Locally-advanced	45 (14.1%)
Metastatic	268 (84.3%)
Brain metastasis	
Yes	111 (34.9%)
No	207 (65.1%)
Line of therapy before crizotinib	
0	16 (5.0%)
1	172 (54.1%)
≥2	130 (40.9%)

MD = missing data; ECOG PS = Eastern Cooperative Oncology Group performance status.

One hundred seventy-two patients (54.1%) received crizotinib as second-line treatment, 16 (5.0%) were treated in the front-line setting, 59 (18.6%) in third-line, and 71 (22.3%) in fourth- or further-line setting. The mean time from diagnosis of advanced disease to initiating crizotinib treatment was 13.9 months (95% CI: 12.4-15.5). Platinum- and pemetrexed-based chemotherapy were administered before crizotinib to 277 (91.7%) and 244 (80.8%) patients, respectively.

### Crizotinib efficacy

Disease progression was observed in 284 patients (89.3%) by the time of data cut-off. The median PFS under crizotinib was 6.8 months (95% CI: 5.6-8.3). Of the 267 evaluable patients, one complete response (0.4%) and 133 partial responses (49.8%) were reported for an ORR of 50.2% (95% CI: 44.2-56.2). The DCR was 74.9% (95% CI: 69.7-80.1). At time of the analysis, 39 patients were still receiving crizotinib.

### Analysis of overall survival with crizotinib

We lost 14 patients (4.4%) to follow-up. By the time of the analysis, 209 patients (65.7%) had died. The median duration of follow-up was 44.4 months (95% CI: 40.6-47.5). The median OS from diagnosis of metastatic disease was 30.9 months (95% CI: 26.7-34.5).

**Figure 2:**
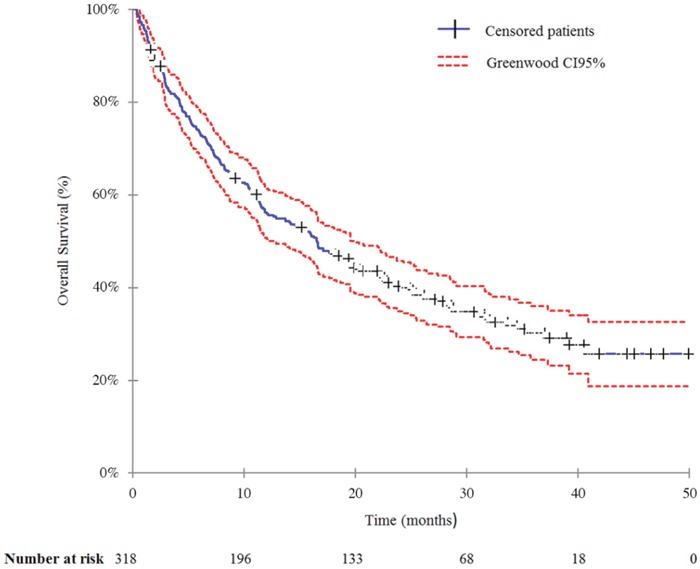
Overall survival from the first crizotinib dose

The median OS from first crizotinib dose was 16.6 months (95% CI: 12.2-19.6) (Figure 2). The 6- and 12-month survival rates were 73.4% (95% CI: 68.5-78.3) and 56.2% (95% CI: 50.7-61.7), respectively. After adjusting for potential confounding factors, multivariable Cox regression (Table 2) revealed former- or never-smoker status on crizotinib initiation, adenocarcinoma histology, and PS 0-1 to be significantly associated with decreased risk of death. Median OS was significantly longer for patients with PS 0-1 than those with PS 2-4 (19.5 months [95% CI: 16.5-25.0] versus 4.5 months [95% CI: 3.0-7.3], log rank p <0.001). The line of crizotinib therapy did not impact OS (Table 2).

**Table 2:** Cox proportional hazard ratio analysis of overall survival from the first crizotinib dose

	Tested	Reference	Univariable analysis		Multivariable analysis	
			HR (95% CI)	p value	HR (95% CI)	p value
Age	< median	≥ median	0.97 (0.74–1.27)	0.81		
Gender	Female	Male	0.99 (0.75–1.29)	0.93		
Smoking status	Never	Former/current	0.79 (0.60–1.03)	0.09	(–)	NS
Current smoker	No	Yes	0.45 (0.30–0.69)	<0.001	0.44 (0.29–0.67)	<0.001
Histology	Adenocarcinoma	Non-adenocarcinoma	0.64 (0.40–1.02)	0.06	0.59 (0.36–0.97)	0.04
PS	0-1	2-4	0.36 (0.27–0.50)	<0.001	0.35 (0.26–0.48)	<0.001
Stage	III	IV	0.84 (0.56–1.26)	0.40		
Brain metastasis	No	Yes	0.95 (0.71–1.25)	0.70		
Number of treatment lines before crizotinib	0-1	≥2	0.79 (0.60–1.04)	0.09	(–)	NS
Setting of administration	EMA approval	EAP	0.86 (0.63–1.17)	0.33		

HR = hazard ratio; CI = confidence interval; PS = performance status; EMA = European Medicines Agency; EAP = expanded access program; NS = not significant.

### Effect on overall survival of systemic treatments following progression on crizotinib

Disease progression on crizotinib was documented in 284 patients. To avoid an immortal time bias and the inclusion in survival analysis of patient who could not receive subsequent treatments after crizotinib, the 21 patients who died under crizotinib were not considered for the survival analysis of systemic treatments following progression on crizotinib. Finally, a population of 263 with documented progressive disease was considered.

The sites of progression are provided in Supplementary Table 1, including 99 cases (37.6%) of cerebral progression, regardless of the extra-cerebral status. There were 136 (51.7%) oligoprogressive diseases, 60 (44.1%) of which occurred in the brain.

CBPD was documented in 86 patients (32.7%). The median duration of CBPD was 6.6 months (range: 0.7-35.2). The baseline characteristics and progression patterns of CBPD and non-CBPD patients are provided in Table 3. Patients under 65 years, with PFS on crizotinib ≥median, cerebral progression, and oligoprogression were more common in the CBPD population. This population was more commonly treated with next-generation ALKis following disease progression. The median OS from the first crizotinib dose was significantly longer in CBPD patients than in non-CBPDs (32.2 months [95% CI: 25.4-NR] versus 11.2 months [95% CI: 8.4-12.9]; log rank p <0.0001), as was median post-PD survival (18.7 months [95% CI: 15.1-26.9] versus 4.0 months [95% CI: 3.0-5.6]; log rank p <0.0001).

**Table 3:** Baseline and post-progression characteristics of the patients who continued crizotinib beyond progressive disease and those who did not

Characteristics	n (%)			*P*-value^a^
	All patients	Continued CBPD	Did not continue CBPD	
	(n=263)	(n=86)	(n=177)	
Age, years				
Median (range)	56.48 (19.2-88.4)	54.83 (19.2-86.8)	58.08 (25.2-88.4)	0.10^b^
<65	194 (73.8)	71 (83)	123 (69.5)	
≥65	69 (26.2)	15 (17)	54 (30.5)	0.02^c^
Gender				
Male	136 (51.7)	49 (57)	87 (49.2)	0.23^c^
Female	127 (48.3)	37 (43)	90 (50.8)	
Ethnicity				
Non-Asian	241 (91.6)	79 (92)	162 (91.5)	0.28^d^
Asian	5 (1.9)	3 (4)	2 (1.1)	
MD	17 (6.5)	4 (6)	13 (7.3)	
Smoking status at baseline				
Current-smoker	26 (10.0)	4 (5)	22 (12.7)	0.09^c^
Former-smoker	88 (34.0)	28 (33)	60 (34.7)	
Never-smoker	145 (56.0)	54 (63)	91 (52.6)	
MD	4	0	4	
Tumour histological type				
Adenocarcinoma	241 (91.6)	83 (96)	158 (89.3)	0.06^d^
Non-adenocarcinoma^d^	22 (8.4)	3 (4)	19 (10.7)	
MD	0	0	0	
PS at baseline				
0-1	192 (78.7)	61 (80)	131 (78.0)	0.69^c^
2-4	52 (21.3)	15 (20)	37 (22.0)	
MD	19	10	9	
Stage at diagnosis				
I/II	3 (1.1)	2 (2)	1 (0.6)	0.16^d^
Metastatic	224 (85.2)	75 (87)	149 (84.2)	
Locally advanced	36 (13.7)	9 (11)	27 (15.3)	
MD	0	0	0	
Brain metastasis at baseline				
Yes	95 (36.1)	31 (36)	64 (36.2)	0.99^c^
No	168 (63.9)	55 (64)	113 (63.8)	
MD	0	0	0	
Lines of therapy before crizotinib				
0-1	157 (59.7)	52 (60)	105 (59.3)	0.86^c^
≥2	106 (40.3)	34 (40)	72 (40.7)	
Cerebral progression				
Yes	99 (37.6)	48 (56)	51 (28.8)	<.001^c^
No	164 (62.4)	38 (44)	126 (71.2)	
Oligoprogression				
Yes	136 (51.7)	61 (71)	75 (42.4)	<.001^c^
No	127 (48.3)	25 (29)	102 (57.6)	
PFS with crizotinib				
≥median	126 (47.9)	59 (69)	67 (37.9)	<.001^c^
<median	137 (52.1)	27 (31)	110 (62.1)	
Subsequent treatment after progression on crizotinib				
Yes	158 (60.1)	50 (58)	108 (61.0)	0.65^c^
No	105 (39.9)	36 (42)	69 (39.0)	
Next-generation ALKis after progression on crizotinib				
Yes	84 (31.9)	36 (41.9)	48 (27.1)	0.02^c^
No	105 (39.9)	50 (58.1)	129 (72.9)	

CBPD = crizotinib beyond progressive disease; MD = missing data; PS = performance status; PFS = progression-free survival; ALKi = ALK inhibitor.

^a^Continued CBPD versus did not continue CBPD.

^b^Student's t-test of comparison of the mean.

^c^Chi-squared test of general association.

^d^Fisher's exact test of general association when sample size requirement for chi-squared test was not matched.

^e^Includes large-cell and other types of carcinoma.

The drugs administered as first-line and second-line post-crizotinib are shown in Table 4 (full details in Supplementary Table 2). The next-generation ALKis administered were ceritinib for 57 patients, alectinib for 19, ceritinib then alectinib for five, ceritinib then lorlatinib in one, and alectinib then ceritinib in two. Crizotinib was rechallenged in nine (3.4%). Chemotherapy was combined with ALKis in eight. Twenty patients received unmonitored subsequent systemic treatment after second-line post-PD: 10 in the group receiving next-generation ALKis and 10 in the group receiving subsequent drugs other than next-generation ALKi.

**Table 4:** Drugs used in first-line and second-line post-disease progression on crizotinib in patients receiving subsequent drugs other than next-generation ALKis and patients receiving next-generation ALKis

Drugs used (n)	First-line post-PD on crizotinib		Second-line post-PD on crizotinib	
	No next-generation ALKis n=74	Next-generation ALKis n=84	No next-generation ALKis n=41	Next-generation ALKis n=42
Chemotherapy (n, %)	49 (66)	12 (14)	25 (61)	14 (33)
Platinum-based	16	3	5	3
Pemetrexed-based	20	7	7	4
Taxane-based	18	4	11	8
Other	7	1	7	2
ALKi (n, %)	10 (14)	69 (82)	11 (27)	24 (57)
Ceritinib	_	49	_	15
Alectinib	_	19	_	7
Lorlatinib	_	_	_	1
Crizotinib	7	1	11	_
Crizotinib and anti-HSP90	3	3	_	1
Chemotherapy and ALKis (n, %)	7 (9)	0 (0)	2 (5)	1 (3)
Others (n, %)	8 (11)	3 (4)	3 (7)	3 (7)
Erlotinib	4	_	3	_
Anti-HSP90	3	3	_	1
Pembrolizumab	_	_	_	2
Anti-MET	1	_	_	_

PD = progressive disease; ALKi = ALK inhibitor.

In order to analyse the effect different systemic treatments have on survival following progression on crizotinib, the patients were separated into three groups: best supportive care (BSC) only (n=105, 40%), subsequent drugs other than next-generation ALKis (n=74, 28.1%), and next-generation ALKis (n=84, 31.9%). The characteristics at baseline and time of disease progression are provided in Table 5. Patients receiving BSC only were older, had poorer PS, were heavily pretreated, and more frequently exhibited PFS on crizotinib <median than those receiving subsequent drugs. Patients receiving next-generation ALKis were younger, more frequently exhibited PFS on crizotinib ≥median, more frequently received CBPD, and more frequently exhibited cerebral progression than patients receiving subsequent drugs other than next-generation ALKis.

**Table 5:** Characteristics at baseline and at time of progression on crizotinib, according to systemic treatment strategies after progression on crizotinib

Characteristics	n (%)		P-value	n (%)		*P*-value
	Best supportive care only	Subsequent drugs		Subsequent drugs other than next-generation ALKis	Next-generation ALKis	
	(n=105)	(n=158)		(n=74)	(n=84)	
**Baseline**						
Age ≥ 65 years	36 (34.3)	33 (20.9)	0.02^b^	21 (28)	12 (14)	0.03^b^
Gender, male	53 (50.5)	83 (52.5)	0.74^b^	40 (54)	43 (51)	0.72^b^
Smoking status			0.12^b^			0.73^b^
Current-smoker	15 (14.3)	11 (7.1)		6 (8)	5 (6)	
Former-smoker	31 (29.5)	57 (37.0)		28 (39)	29 (35)	
Never-smoker	59 (56.2)	86 (55.9)		38 (53)	48 (59)	
Non-adenocarcinoma histology^d^	12 (11.4)	10 (6.3)	0.14^b^	8 (11)	2 (2)	0.05^c^
PS 2-4	37 (35.3)	15 (9.5)	<0.001^b^	10 (14)	5 (7)	0.17^b^
Metastatic stage	90 (85.7)	134 (84.8)	0.99^c^	62 (84)	72 (86)	0.85^c^
Brain metastasis	33 (31.4)	62 (39.2)	0.20^b^	26 (35)	36 (43)	0.32^b^
PFS with crizotinib <median	75 (71.4)	62 (39.2)	<0.001^b^	40 (54)	22 (26)	<0.001^b^
≥2 lines before crizotinib	52 (49.5)	54 (34.2)	0.01^b^	27 (37)	27 (32)	0.57^b^
**At time of progression on crizotinib**						
Cerebral progression	38 (36.2)	61 (38.6)	0.69^d^	20 (27)	41 (49)	0.005^b^
Oligoprogression	57 (54.3)	79 (50.0)	0.50^b^	38 (51)	41 (49)	0.75^b^
CBPD						
Yes	36 (34.3)	50 (31.6)	0.65^b^	14 (19)	36 (43)	0.001^b^
Median duration (days)	101	79.5	0.36^a^	44.5	142.5	0.10^a^

ALKi = ALK inhibitor; PS = performance status; CBPD = crizotinib beyond progressive disease; PFS = progression-free survival.

^a^Student's t-test of comparison of the mean.

^b^Chi-squared test of general association.

^c^Fisher's exact test of general association when sample size requirement for chi-squared test was not matched.

^d^Includes large-cell and other types of carcinoma.

Median post-PD survival was 6.5 months (95% CI: 5.3-9.8). Multivariable Cox regression (Table 6) revealed that next-generation ALKis and CBPD were associated with improved survival from the first crizotinib dose, as well as improved post-PD survival, as were baseline PS 0-1, PFS with crizotinib ≥median, cerebral progression, and oligoprogression. Subsequent treatment other than next-generation ALKis did not improve survival. BSC only was associated with worse survival from the first crizotinib dose and worse post-PD survival.

**Table 6:** Cox multivariate analysis* of survival from the first crizotinib dose and post-progressive disease survival in the population of patients with documented progressive disease on crizotinib (n=263)

Variable	Tested	Reference	Multivariable analysis			
			Survival from the first crizotinib dose		Survival post-PD on crizotinib	
			HR (95% CI)	p value	HR (95% CI)	p value
PS	0-1	2-4	0.49 (0.34–0.70)	p<0.0001	0.43 (0.30–0.62)	p<0.0001
PFS with crizotinib	≥median	<median	0.28 (0.20–0.40)	p<0.0001	0.68 (0.48–0.95)	0.02
Cerebral progression	Yes	No	0.55 (0.39–0.77)	0.0006	0.67 (0.49–0.94)	0.02
Oligoprogression	Yes	No	0.63 (0.46–0.87)	0.005	0.60 (0.44–0.83)	0.002
Crizotinib beyond PD	Yes	No	0.52 (0.35–0.77)	0.001	0.46 (0.31–0.68)	p<0.0001
Systemic treatment after progression on crizotinib:						
BSC only	Yes	No	2.06 (1.45–2.93)	<0.0001	2.39 (1.67–3.42)	<0.0001
Subsequent systemic treatment but no next- generation ALKis	Yes	No	(–)	NS	(–)	NS
Next-generation ALKis	Yes	No	0.34 (0.21–0.55)	<0.0001	0.36 (0.23–0.57)	<0.0001

PD = progressive disease; HR = hazard ratio; CI = confidence interval; PS = performance status; PFS = progression-free survival; BSC = best supportive care; ALKi = ALK inhibitor; NS = not significant.

*The full Cox univariate and multivariate proportional hazard ratio analysis is provided in Supplementary Table 3 and Supplementary Table 4.

**Figure 3:**
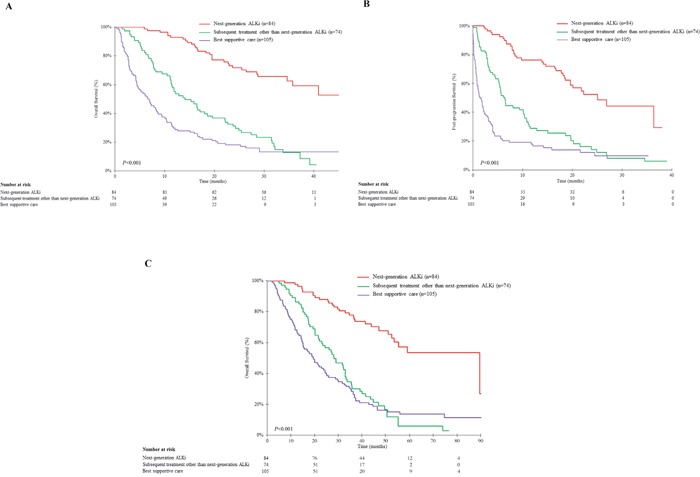
Overall survival according to subsequent systemic treatments initiated after progression on crizotinib in patients with documented progressive disease on crizotinib (n=263) **A**. Overall survival from the first crizotinib dose and **B**. survival post-progressive disease on crizotinib for the 84 patients receiving next-generation ALK inhibitors after progression on crizotinib, compared with the 74 patients receiving subsequent treatments other than next-generation ALK inhibitors and the 105 patients receiving best supportive care only. **C**. Overall survival from the diagnosis of metastatic disease in the 84 patients receiving next-generation ALK inhibitors after progression on crizotinib.

For patients receiving next-generation ALKis, median OS from the first crizotinib dose was not reached (Figure 3A). The 1- and 3-year survival rates from the first crizotinib dose were 92.9% (95% CI: 87.3-98.4) and 59.2% (95% CI: 46.4-72.1), respectively. Post-PD survival was significantly longer for patients receiving next-generation ALKis than either those receiving subsequent drugs other than next-generation ALKis or those receiving BSC only (25.0 months [95% CI: 18.6-NR] versus 6.4 months [95% CI: 5.1-10.2] versus 1.5 [95% CI: 0.8-2.1], log rank p=0.0002) (Figure 3B).

The median OS from diagnosis of metastatic disease was 89.6 months (95% CI: 53.5-not reached) for patients receiving next-generation ALKi and significantly longer than either those receiving subsequent drugs other than next-generation ALKi (28.2 months [95% CI: 22.1-33.0]) or those receiving BSC only (19.6 months [95% CI: 15.1-24.5]) (log rank *P*<0.001) (Figure 3C).

## DISCUSSION

Our findings provide a robust OS estimation for patients receiving crizotinib for advanced *ALK*-positive NSCLC primarily pretreated with first-line platinum-based regimens. Additionally, our analysis of systemic treatments following disease progression on crizotinib suggests that next-generation ALKis substantially prolong survival after crizotinib failure in comparison with other treatment strategies.

We report a median OS of 16.6 months after initiation of crizotinib, which is slightly shorter than the previous estimation of 21.7 months reported by the PROFILE 1007 trial evaluating crizotinib in the second-line setting. This could be the result of selecting patients in clinical trials compared to all-comer patients treated in daily practice. Our patients were older, less commonly women and non-smokers, with poorer PS than those enrolled in PROFILE 1007 [4]. In 40.9% of cases, patients received more than one systemic treatment before crizotinib and were less able to receive subsequent treatments, though the line of crizotinib treatment did not impact survival outcomes on crizotinib in our study. Almost all were of non-Asian ethnicity, compared to 54% of the patients in PROFILE 1007 [4]. A non-Asian ethnicity-related negative effect on outcome could not be excluded [23].

Current smoking at time of crizotinib initiation had a strong negative effect on survival outcomes. *ALK* rearrangement is detected in higher proportions in non-smokers and former smokers [1]. Very few current smokers were enrolled in clinical trials with crizotinib, meaning little is known about crizotinib efficacy in this patient subset [3, 4]. The current-smoker population could consist of those with very poor prognosis and a specific biological pattern, explaining the lack of efficacy of crizotinib. Furthermore, cigarette smoking induces cytochromes CYP1A1/1A2 and is hypothesized to alter anti-EGFR erlotinib pharmacokinetics, resulting in worse clinical outcomes [24, 25]. Crizotinib elimination via CYP1A1/1A2 has not been reported, yet our data suggests cigarette smoking has a potential impact on its pharmacokinetics [26, 27]. Nevertheless, only 29 patients were current smokers at time of crizotinib initiation in our study. Our results warrant validation in a larger cohort. On the other hand, PS 2-4 at time of crizotinib initiation was associated with worse survival with crizotinib and after disease progression. This suggests that ALKis should be given to *ALK*-positive patients as soon as possible in the disease course.

CBPD was associated with remarkably similar survival outcomes to those previously reported by Ou *et al*. [9]. CLINALK and Ou *et al*. studies reported median OS with crizotinib of 32.2 and 29.6 months in the CBPD population and 11.2 and 10.8 months in non-CBPD, respectively [9]. The similar survival benefit we observed with CBPD in two independent cohorts, and persistence of this benefit following adjustment for the different systemic treatment strategies initiated after disease progression on crizotinib, confirm the validity of this approach, previously legitimised by the lack of effective and well-tolerated drugs available for strategies following crizotinib failure. Next-generation ALKis have emerged as the preferred treatment in this setting due to their ability to overcome crizotinib resistance. In our study, they deeply impacted survival outcomes when given after disease progression on crizotinib, whereas other systemic treatments did not and, as expected, BSC only was associated with worse survival.

Given its retrospective nature, this survival analysis had several limitations. We included patients receiving crizotinib at different times in their disease course, in second- or third-line in 72.7% of the cases. The survival analysis focused on three non-randomized and unmatched groups of patients according to systemic treatment received after disease progression on crizotinib. As a result, the characteristics of these three groups are partially imbalanced, with biases in patient selection potentially causing the improved survival observed in those receiving CBPD or next-generation ALKis. Alternatively, this could also be due to specific tumour biology and high sensitivity to ALK inhibition, rather than a direct effect of treatment strategies. Patients received next-generation ALKis as clinical trial participants, all with good prognosis. Finally, locally-ablative treatments in cases of oligoprogressive disease were not recorded, potentially causing a partial bias in our results.

Nevertheless, we provide an estimation of survival benefit with next-generation ALKis adjusted for potential confounding factors, including patterns of progression on crizotinib and CBPD, compared to a population treated with systemic treatments other than next-generation ALKis or BSC only. A recently published retrospective analysis of 73 patients treated with crizotinib then ceritinib reported a 49.4-month OS from diagnosis of metastatic disease, though did not provide a comparator population [20]. Two smaller studies with 11 and 13 patients treated with crizotinib then reported good survival outcomes [21, 22]. In our study, the 84 patients receiving next-generation ALKis after crizotinib achieved an 89.6-month OS from diagnosis of metastatic disease, with a 59.2% 3-year survival from the first crizotinib dose and median post-PD survival of 25.0 months. The survival rates reported here in a large population treated with crizotinib then next-generation ALKis could represent an interesting benchmark for ongoing clinical trials assessing how best to sequence the available ALKis. The ALEX trial (NCT02075840) is comparing crizotinib and alectinib in first-line setting with no crossover at time of progression. In contrast, the NCI ALK Master Protocol (NCT02465060) will compare in first-line a standard treatment arm with crizotinib to several next-generation ALKis and evaluate different sequential strategies by incorporating systematic crossover in each treatment arm.

In conclusion, the prolonged survival observed in crizotinib clinical trials in *ALK*-positive NSCLC can also be observed in less selective patient populations treated in routine practice. The remarkable median survival from diagnosis of metastatic disease of 89.6 months reported in patients treated with next-generation ALKis after crizotinib emphasizes the importance of accelerated access to diagnostic tools and targeted therapy in molecularly-defined populations. While this study was unable to resolve the question of how best to sequence ALKis, it could provide a rationale that supports the use of sequential ALKis rather than non-targeted treatment after crizotinib failure, along with offering an historical benchmark for the ongoing clinical trials in this setting.

## MATERIALS AND METHODS

### Study population and procedures

This retrospective study included only patients with diagnosis of *ALK*-rearranged NSCLC determined by fluorescent *in situ* hybridization (FISH, performed on a routine basis at certified molecular genetics French National Cancer Institute [INCa] platforms using a certified break-apart FISH assay), with advanced/metastatic NSCLC, aged ≥18 years, not enrolled in a crizotinib trial, having received at least 7 days of crizotinib treatment. All received 250mg oral crizotinib twice daily at initiation.

The French crizotinib expanded access program (EAP) enrolled 313 patients exhibiting any *ALK*-positive tumours from November 18^th^ 2010 to October 23^th^ 2012. The EAP database was provided by Pfizer. Of the 117 identified investigational centres, 80 agreed to participate. After EAP discontinuation, we enrolled patients receiving second-line crizotinib as approved drug until December 31^th^ 2013 at participating centres.

Data and survival follow-up were extracted from medical records by investigators in each centre and documented in a standard case report form. Database is held by the French Collaborative Thoracic Intergroup (IFCT) that ensured the quality of the data collected by monitoring the centres via periodic visits of IFCT clinical research associates. Medical monitoring was performed by two co-authors (MD, DMS). The source documents proving the collected data's integrity are filed at the investigational centre.

### Definitions and study endpoints

The sites where PD manifested were reported. Oligoprogressive disease was defined as progression in only one site. CBPD was defined as continuing crizotinib for over 21 days following RECIST-defined PD and best response to crizotinib other than PD. First-line and second-line drugs following crizotinib failure and corresponding response according to RECIST 1.1. were monitored. Crizotinib rechallenge was defined as crizotinib initiation following at least one systemic therapy following PD under crizotinib [28].

The primary end-point was OS measured from the date of first crizotinib dose. Secondary endpoints included: objective response rate (ORR) according to RECIST 1.1, evaluated by investigators; disease control rate (DCR); PFS, according to RECIST 1.1.; OS from PD under crizotinib (post-PD survival); OS from diagnosis of metastatic disease.

### Study oversight

This non-interventional study was conducted in accordance with the Declaration of Helsinki and Good Clinical Practice guidelines, approved by a national ethics committee, French Advisory Committee on Information Processing in Material Research in the Field of Health, and France's national data protection authority (CNIL). All participating departments approved the study protocol. All included patients still alive received information from their referring physician.

### Statistical analysis

Variable characteristics were compared with the chi-squared or Fisher's exact tests for qualitative variables and Student's t-test or ANOVA for quantitative variables. The Kaplan-Meier method was used to estimate all OS endpoints. We estimated hazard ratios (HRs) and 95% confidence intervals (CIs) using a Cox model. Univariate Cox models were applied to select the most promising prognostic variables (threshold p=0.20). A multivariate Cox model was then applied using a backwards procedure to adjust for potential confounders. OS was defined as the date of first crizotinib dose to death or final follow-up. Post-PD survival was defined as the date of RECIST-defined PD under crizotinib to death or final follow-up. The cut-off for survival analysis was July 31^st^ 2015. All statistical tests were two-sided, and a p value <0.05 was deemed statistically significant. All analyses were performed using SAS software, Version 9.3 (SAS Institute).

We wish to thank the following individuals for their participation in data collection, monitoring, and computing: S Dos Santos and A Lejeune (*Intergroupe Francophone de Cancérologie Thoracique*, Paris, France).

## SUPPLEMENTARY MATERIALS FIGURES AND TABLES



## References

[1] Soda M, Choi YL, Enomoto M, Takada S, Yamashita Y, Ishikawa S, Fujiwara S, Watanabe H, Kurashina K, Hatanaka H, Bando M, Ohno S, Ishikawa Y (2007). Identification of the transforming EML4-ALK fusion gene in non-small-cell lung cancer. Nature.

[2] Barlesi F, Mazieres J, Merlio J-P, Debieuvre D, Mosser J, Lena H, Ouafik L, Besse B, Rouquette I, Westeel V, Escande F, Monnet I, Lemoine A (2016). Routine molecular profiling of patients with advanced non-small-cell lung cancer: results of a 1-year nationwide programme of the French Cooperative Thoracic Intergroup (IFCT). Lancet.

[3] Solomon BJ, Mok T, Kim D-W, Wu Y-L, Nakagawa K, Mekhail T, Felip E, Cappuzzo F, Paolini J, Usari T, Iyer S, Reisman A, Wilner KD (2014). First-line crizotinib versus chemotherapy in ALK-positive lung cancer. N Engl J Med.

[4] Shaw AT, Kim D-W, Nakagawa K, Seto T, Crinó L, Ahn M-J, De Pas T, Besse B, Solomon BJ, Blackhall F, Wu Y-L, Thomas M, O’Byrne KJ (2013). Crizotinib versus chemotherapy in advanced ALK-positive lung cancer. N Engl J Med.

[5] Shaw AT, Yeap BY, Solomon BJ, Riely GJ, Gainor J, Engelman JA, Shapiro GI, Costa DB, Ou S-HI, Butaney M, Salgia R, Maki RG, Varella-Garcia M (2011). Effect of crizotinib on overall survival in patients with advanced non-small-cell lung cancer harbouring ALK gene rearrangement: a retrospective analysis. Lancet Oncol.

[6] Shaw AT, Janne PA, Besse B, Solomon BJ, Blackhall FH, Camidge RD, Mok T, Hirsh V, Scranton JR, Polli A, Tang Y, Wilner KD, Kim DW (2016). Crizotinib vs chemotherapy in ALK+ advanced non-small cell lung cancer (NSCLC): Final survival results from PROFILE 1007. J Clin Oncol.

[7] Weickhardt AJ, Scheier B, Burke JM, Gan G, Lu X, Bunn PA, Aisner DL, Gaspar LE, Kavanagh BD, Doebele RC, Camidge DR (2012). Local ablative therapy of oligoprogressive disease prolongs disease control by tyrosine kinase inhibitors in oncogene-addicted non-small-cell lung cancer. J Thorac Oncol.

[8] Gan GN, Weickhardt AJ, Scheier B, Doebele RC, Gaspar LE, Kavanagh BD, Camidge DR (2014). Stereotactic radiation therapy can safely and durably control sites of extra-central nervous system oligoprogressive disease in anaplastic lymphoma kinase-positive lung cancer patients receiving crizotinib. Int J Radiat Oncol Biol Phys.

[9] Ou S-HI, Jänne PA, Bartlett CH, Tang Y, Kim D-W, Otterson GA, Crinò L, Selaru P, Cohen DP, Clark JW, Riely GJ (2014). Clinical benefit of continuing ALK inhibition with crizotinib beyond initial disease progression in patients with advanced ALK-positive NSCLC. Ann Oncol.

[10] Browning ET, Weickhardt AJ, Camidge DR (2013). Response to crizotinib rechallenge after initial progression and intervening chemotherapy in ALK lung cancer. J Thorac Oncol.

[11] Matsuoka H, Kurata T, Okamoto I, Kaneda H, Tanaka K, Nakagawa K (2013). Clinical response to crizotinib retreatment after acquisition of drug resistance. J Clin Oncol.

[12] Berge EM, Lu X, Maxson D, Barón AE, Gadgeel SM, Solomon BJ, Doebele RC, Varella-Garcia M, Camidge DR (2013). Clinical benefit from pemetrexed before and after crizotinib exposure and from crizotinib before and after pemetrexed exposure in patients with anaplastic lymphoma kinase-positive non-small-cell lung cancer. Clin Lung Cancer.

[13] Shaw AT, Kim D-W, Mehra R, Tan DSW, Felip E, Chow LQM, Camidge DR, Vansteenkiste J, Sharma S, De Pas T, Riely GJ, Solomon BJ, Wolf J (2014). Ceritinib in ALK-rearranged non-small-cell lung cancer. N Engl J Med.

[14] Kim D-W, Mehra R, Tan DSW, Felip E, Chow LQM, Camidge DR, Vansteenkiste J, Sharma S, De Pas T, Riely GJ, Solomon BJ, Wolf J, Thomas M (2016). Activity and safety of ceritinib in patients with ALK-rearranged non-small-cell lung cancer (ASCEND-1): updated results from the multicentre, open-label, phase 1 trial. Lancet Oncol.

[15] Gadgeel SM, Gandhi L, Riely GJ, Chiappori AA, West HL, Azada MC, Morcos PN, Lee R-M, Garcia L, Yu L, Boisserie F, Di Laurenzio L, Golding S (2014). Safety and activity of alectinib against systemic disease and brain metastases in patients with crizotinib-resistant ALK-rearranged non-small-cell lung cancer (AF-002JG): results from the dose-finding portion of a phase 1/2 study. Lancet Oncol.

[16] Shaw AT, Gandhi L, Gadgeel S, Riely GJ, Cetnar J, West H, Camidge DR, Socinski MA, Chiappori A, Mekhail T, Chao BH, Borghaei H, Gold KA (2016). Alectinib in ALK-positive, crizotinib-resistant, non-small-cell lung cancer: a single-group, multicentre, phase 2 trial. Lancet Oncol.

[17] Ou S-HI, Ahn JS, De Petris L, Govindan R, Yang JC-H, Hughes B, Lena H, Moro-Sibilot D, Bearz A, Ramirez SV, Mekhail T, Spira A, Bordogna W (2016). Alectinib in Crizotinib-Refractory ALK-Rearranged Non-Small-Cell Lung Cancer: A Phase II Global Study. J Clin Oncol.

[18] Zou HY, Friboulet L, Kodack DP, Engstrom LD, Li Q, West M, Tang RW, Wang H, Tsaparikos K, Wang J, Timofeevski S, Katayama R, Dinh DM (2015). PF-06463922, an ALK/ROS1 Inhibitor, Overcomes Resistance to First and Second Generation ALK Inhibitors in Preclinical Models. Cancer Cell.

[19] Gettinger SN, Bazhenova LA, Langer CJ, Salgia R, Gold KA, Rosell R, Shaw AT, Weiss GJ, Tugnait M, Narasimhan NI, Dorer DJ, Kerstein D, Rivera VM (2016). Activity and safety of brigatinib in ALK-rearranged non-small-cell lung cancer and other malignancies: a single-arm, open-label, phase 1/2 trial. Lancet Oncol.

[20] Gainor JF, Tan DSW, De Pas T, Solomon BJ, Ahmad A, Lazzari C, de Marinis F, Spitaleri G, Schultz K, Friboulet L, Yeap BY, Engelman JA, Shaw AT (2015). Progression-Free and Overall Survival in ALK-Positive NSCLC Patients Treated with Sequential Crizotinib and Ceritinib. Clin Cancer Res.

[21] Chiari R, Metro G, Iacono D, Bellezza G, Rebonato A, Dubini A, Sperduti I, Bennati C, Paglialunga L, Burgio MA, Baglivo S, Giusti R, Minotti V (2015). Clinical impact of sequential treatment with ALK-TKIs in patients with advanced ALK-positive non-small cell lung cancer: Results of a multicenter analysis. Lung Cancer.

[22] Ito K, Hataji O, Kobayashi H, Fujiwara A, Yoshida M, D’Alessandro-Gabazza CN, Itani H, Tanigawa M, Ikeda T, Fujiwara K, Fujimoto H, Kobayashi T, Gabazza EC (2017). Sequential Therapy with Crizotinib and Alectinib in ALK-Rearranged Non-Small Cell Lung Cancer-A Multicenter Retrospective Study. J Thorac Oncol.

[23] Tan DSW, Mok TSK, Rebbeck TR (2016). Cancer Genomics: Diversity and Disparity Across Ethnicity and Geography. J Clin Oncol.

[24] Hamilton M, Wolf JL, Rusk J, Beard SE, Clark GM, Witt K, Cagnoni PJ (2006). Effects of smoking on the pharmacokinetics of erlotinib. Clin Cancer Res.

[25] Hughes AN, O’Brien MER, Petty WJ, Chick JB, Rankin E, Woll PJ, Dunlop D, Nicolson M, Boinpally R, Wolf J, Price A (2009). Overcoming CYP1A1/1A2 mediated induction of metabolism by escalating erlotinib dose in current smokers. J Clin Oncol.

[26] Hamilton G, Rath B, Burghuber O (2015). Pharmacokinetics of crizotinib in NSCLC patients. Expert Opin Drug Metab Toxicol.

[27] Wang E, Nickens DJ, Bello A, Khosravan R, Amantea M, Wilner K, Parivar K, Tan W (2016). Clinical Implications of the Pharmacokinetics of Crizotinib in Populations of Patients with Non-Small Cell Lung Cancer. Clin Cancer Res.

[28] Kuczynski EA, Sargent DJ, Grothey A, Kerbel RS (2013). Drug rechallenge and treatment beyond progression--implications for drug resistance. Nat Rev Clin Oncol.

