# Supplementary material for: Overall survival with crizotinib and next-generation ALK inhibitors in ALK-positive non-small-cell lung cancer (IFCT-1302 CLINALK): a French nationwide cohort retrospective study
Source: Oncotarget. 2017 Feb 26;8(13):21903–17. doi: 10.18632/oncotarget.15746 (PMC5400633; doi:10.18632/oncotarget.15746)
Supplement: Supplementary file 1 [file oncotarget-08-21903-s001.pdf]

Overall survival with crizotinib and next-generation ALK inhibitors in *ALK*-positive non-small-cell lung cancer (IFCT-1302 CLINALK): a French nationwide cohort retrospective study

SUPPLEMENTARY MATERIALS

SUPPLEMENTARY TABLES

Supplementary Table 1: Sites of disease progression in patients with documented progressive disease (n=263) and those presenting with oligoprogression (n=136)

Site of disease progression	Patients with documented progressive disease n=263 (n, %)	Patients with oligoprogression n=136 (n, %)
Brain	99 (37.6)	60 (44.1)
Primary tumour	83 (31.6)	17 (12.5)
Liver	55 (20.9)	13 (9.6)
Regional lymph nodes	47 (17.9)	1 (0.7)
Bone	40 (15.2)	12 (8.8)
Lung	37 (14.1)	8 (5.9)
Pleural effusion	37 (14.1)	7 (5.1)
Distant lymph nodes	22 (8.4)	2 (1.5)
Adrenal gland	20 (7.6)	4 (2.9)
Peritoneal	9 (3.4)	4 (2.9)
Carcinomatous meningitis	6 (2.3)	4 (2.9)
Choroidal / retinal	3 (1.1)	2 (1.5)
Cutaneous	2 (0.8)	0 (0)
Renal	2 (0.8)	1 (0.7)
Thoracic wall	2 (0.8)	0 (0)
Splenic	1 (0.4)	0 (0)

Supplementary Table 2: Drugs used in first-line and second-line post-progressive disease on crizotinib and details of response

Drugs used	First-line post-crizotinib n=158	Objective response CR/PR/SD/PD/NE/MD	Second-line post-crizotinib n=83 (n, %)	Objective response CR/PR/SD/PD/NE/MD
Chemotherapy (n, %)	61 (38.6)		39 (47)	
Pemetrexed	18	0 / 0 / 10 / 6 / 2 / 0	6	0 / 2 / 0 / 3 / 0 / 1
Pemetrexed/carboplatin	8	0 / 2 / 4 / 1 / 1 / 0	4	0 / 1 / 1 / 1 / 1 / 0
Pemetrexed/cisplatin	1	0 / 1 / 0 / 0 / 0 / 0	1	0 / 0 / 0 / 0 / 1 / 0
Pemetrexed/carboplatin/ bevacizumab	3	0 / 2 / 1 / 0 / 0 / 0	_	_
Docetaxel	13	0 / 1 / 4 / 7 / 1 / 0	9	0 / 1 / 1 / 4 / 2 / 1
Paclitaxel	1	0 / 0 / 0 / 0 / 1 / 0	3	0 / 0 / 0 / 2 / 1 / 0
Paclitaxel/carboplatin	5	0 / 1 / 1 / 2 / 1 / 0	3	0 / 0 / 0 / 3 / 0 / 0
Paclitaxel/carboplatin/bevacizumab	1	0 / 0 / 0 / 0 / 1 / 0	_	_
Paclitaxel/bevacizumab	2	0 / 0 / 1 / 1 / 0 / 0	3	0 / 0 / 0 / 2 / 1 / 0
Paclitaxel/trastuzumab	_	_	1	0 / 0 / 1 / 0 / 0 / 0
Etoposide/cisplatin	1	0 / 0 / 0 / 1 / 0 / 0	_	_
Gemcitabine	4	0 / 1 / 0 / 3 / 0 / 0	6	0 / 0 / 0 / 5 / 1 / 0
Navelbine	2	0 / 0 / 0 / 1 / 1 / 0	3	0 / 0 / 0 / 0 / 2 / 1
Methotrexate	1	0 / 0 / 0 / 1 / 0 / 0	_	_
Unknown	1	0 / 0 / 0 / 0 / 0 / 1	_	_
ALK inhibitors	82 (51.9)		35 (42)	
Ceritinib	49	0 / 20 / 11 / 6 / 2 / 10	15	1 / 8 / 1 / 1 / 2 / 2
Alectinib	19	0 / 12 / 1 / 5 / 1 / 0	7	0 / 3 / 2 / 1 / 0 / 1
Lorlatinib	_	_	1	_
Crizotinib	8	0 / 3 / 2 / 2 / 1 / 0	11	0 / 3 / 1 / 4 / 3 / 0
Crizotinib and anti-HSP90	6	_	1	_
Chemotherapy and ALK inhibitors	7 (4.4)		3 (4)	
Crizotinib/pemetrexed	2	0 / 1 / 0 / 1 / 0 / 0	2	0 / 0 / 1 / 1 / 0 / 0
Crizotinib/pemetrexed/carboplatin	1	0 / 0 / 1 / 0 / 0 / 0	_	_
Crizotinib/paclitaxel	1	0 / 0 / 1 / 0 / 0 / 0	_	_
Crizotinib/docetaxel/carboplatin	1	0 / 1 / 0 / 0 / 0 / 0	_	_
Crizotinib/docetaxel	1	0 / 0 / 1 / 0 / 0 / 0	_	_
Crizotinib/oxaliplatin	1	0 / 1 / 0 / 0 / 0 / 0	_	_
Ceritinib/paclitaxel//bevacizumab	_	_	1	0 / 0 / 1 / 0 / 0 / 0
Others	8 (5.1)		6 (7)	
Erlotinib	4	0 / 0 / 0 / 1 / 1 / 2	3	0 / 0 / 0 / 2 / 1 / 0
Anti-HSP90	3	_	1	_
Anti-MET	1	_	_	_
Pembrolizumab	_	_	2	_

CR = complete response; PR = partial response; SD = stable disease; PD = progressive disease; NE = not evaluable; MD = missing data.

Supplementary Table 3: Impact on survival of the post-progression systemic treatments used (n=263): Cox proportional hazard ratio analysis of post-progression survival

Variable	Tested	Reference	Univariable analysis		Multivariable analysis	
			HR (95% CI)	p value	HR (95% CI)	p value
Age	≥median	<median	1.09 (0.81–1.45)	0.57	(–)	
Gender	Female	Male	1.12 (0.84–1.49)	0.45	(–)	
Smoking status	Never	Former/current	0.96 (0.72–1.28)	0.77	(–)	
Current smoker	No	Yes	0.51 (0.33-0.79)	0.003	(–)	NS
Histology	Adenocarcinoma	Non-adenocarcinoma	0.64 (0.40–1.03)	0.06	(–)	NS
PS	0-1	2-4	0.37 (0.26–0.52)	<0.0001	0.43 (0.30–0.62)	p<0.0001
Stage	IV	III	0.97 (0.63-1.50)	0.90		
Line of therapy before crizotinib	0-1	≥2	0.84 (0.63–1.13)	0.25	(–)	
PFS with crizotinib	≥median	<median	0.37 (0.27–0.49)	<0.0001	0.68 (0.48–0.95)	0.02
Cerebral progression	Yes	No	0.48 (0.35–0.66)	<0.0001	0.67 (0.49–0.94)	0.02
Oligoprogression	Yes	No	0.61 (0.46–0.82)	<0.001	0.60 (0.44–0.83)	0.002
Crizotinib beyond PD	Yes	No	0.39 (0.28–0.55)	<0.0001	0.46 (0.31–0.68)	p<0.0001
Best supportive care only after progression on crizotinib	Yes	No	3.17 (2.36–4.25)	<0.0001	2.39 (1.67–3.42)	<0.0001
Subsequent systemic treatment other than next-generation ALKis after progression on crizotinib	Yes	No	1.22 (0.90–1.66)	0.19	(–)	NS
Next-generation ALKis after progression on crizotinib	Yes	No	0.24 (0.17–0.35)	<0.0001	0.36 (0.23–0.57)	<0.0001

HR = hazard ratio; CI = confidence interval; PS = performance status; PFS = progression-free survival; PD = progressive disease; ALKi = ALK inhibitor; NS = not significant.

Supplementary Table 4: Impact on survival of the post-progression systemic treatment options used (n=263): Cox proportional hazard ratio analysis of survival from the first crizotinib dose

Variable	Tested	Reference	Univariable analysis		Multivariable analysis	
			HR (95% CI)	p value	HR (95% CI)	p value
Age	≥ median	< median	1.01 (0.75–1.35)	0.95	(–)	
Gender	Female	Male	1.02 (0.77–1.37)	0.87	(–)	
Smoking status	Never	Former/current	0.86 (0.64–1.15)	0.32	(–)	
Current smoker	No	Yes	0.41 (0.27-0.64)	<0.0001	(–)	NS
Histology	Adenocarcinoma	Non-adenocarcinoma	0.49 (0.30–0.78)	0.003	(–)	NS
PS	0-1	2-4	0.40 (0.28–0.56)	<0.0001	0.49 (0.34–0.70)	p<0.0001
Stage	IV	III	0.99 (0.64-1.52)	0.97		
Line of therapy before crizotinib	0-1	≥2	0.83 (0.62–1.11)	0.21	(–)	
PFS with crizotinib	≥median	<median	0.21 (0.16–0.29)	<0.0001	0.28 (0.20–0.40)	p<0.0001
Cerebral progression	Yes	No	0.45 0.32–0.62)	<0.0001	0.55 (0.39–0.77)	0.0006
Oligoprogression	Yes	No	0.64 (0.47–0.86)	0.003	0.63 (0.46–0.87)	0.005
Crizotinib beyond PD	Yes	No	0.37 (0.26–0.53)	<0.0001	0.52 (0.35–0.77)	0.001
Best supportive care only after progression on crizotinib	Yes	No	2.92 (2.17–3.92)	<0.0001	2.06 (1.45–2.93)	<0.0001
Subsequent systemic treatment other than next-generation ALKis after progression on crizotinib	Yes	No	1.40 (1.03–1.91)	0.03	(–)	NS
Next-generation ALKis after progression on crizotinib	Yes	No	0.21 (0.14–0.31)	<0.0001	0.34 (0.21–0.55)	<0.0001

HR = hazard ratio; CI = confidence interval; PS = performance status; PFS = progression-free survival; PD = progressive disease; ALKi = ALK inhibitor; NS = not significant.
